# Editorial: Update on the Diagnosis and Management of CIDP Variants

**DOI:** 10.3389/fneur.2021.796841

**Published:** 2021-11-24

**Authors:** Elisabeth Chroni, Kleopas A. Kleopa

**Affiliations:** ^1^Department of Neurology, University of Patras, Patras, Greece; ^2^Center for Neuromuscular Disorders and Department of Neuroscience, The Cyprus Institute of Neurology and Genetics, Engomi, Cyprus

**Keywords:** chronic inflammatory polyneuropathy, clinical manifestations, auto-immune profile, biomarkers, treatment

Chronic inflammatory demyelinating polyneuropathy (CIDP) is a potentially treatable condition with great variability in terms of clinical presentation, pathogenesis, auto-antibody profile, disease progression, and response to treatment. The recognition of several CIDP variants within the spectrum of CIDP, such as distal acquired symmetric neuropathy (DADS), multifocal acquired demyelinating sensory and motor neuropathy (MADSAM), focal CIDP forms, pure sensory and pure motor CIDP, and chronic immune sensory polyradiculopathy (CISP), emphasizes the need for accurate diagnosis and appropriate treatment.

Over the last decade, the presence of several auto-antibodies against peripheral nerve targets has been reported in a minority of patients with CIDP. Some of them, such as neurofascin 155/186 or contactin 1 antibodies, have been associated with distinct clinical presentations and response to treatment. In cohorts of CIDP patients, the detection of different antibodies targeting nerve antigens and their clinical syndromes are currently under study. However, limited evidence is available in regards to optimal management, clinical course, and outcome of atypical CIDP. There are reports suggesting that not all CIDP sub-types respond equally well to first-line treatment, while novel immunomodulatory strategies have not been designed for CIDP variants.

Currently, several potential biomarkers for quantitative evaluation of disease progression are under investigation in order to decide upon appropriate therapeutic approaches. Stascheit et al. investigated calprotectin (CLP), a calcium-binding protein of the S100 family, which triggers signaling pathways involved in inflammatory processes as an index of CIDP activity. These authors performed a pilot cross-sectional case-control study of 63 patients with CIDP (46% CIDP variants) and showed that CLP and serum neurofilament light chain (sNfl) levels were significantly higher than those in 40 healthy controls. In the CIDP cohort, CLP was related to active inflammatory course assessed by CIDP disease activity scale (CDAS) and Medical Research Council (MRC)-sum score. On the other hand, sNfl levels were not correlated with CDAS but were associated with the severity of disease deficits assessed by MRC-sum score, inflammatory neuropathy cause and treatment disability score, grip strength, and walking distance. The clinical significance of these biomarkers should be further explored in larger studies allowing subgroup analysis.

Diagnosis of CIDP and CIDP variants in children is not an easy task, since these disorders are rarer than in adults and require differentiation from the hereditary demyelinating neuropathies that are by far more common in childhood. Lukawska et al. retrospectively reviewed medical records of 37 children divided into age subgroups (under 4, 4–13, and 13–18 years) with CIDP from a single center. The diagnosis was delayed by more than 1 year in 53.6% of children under the age of 13 years, implying difficulties in documentation of CIDP in this population. Noticeable findings were acute onset (<8 weeks) in 16.2% of patients, the symmetrical weakness of proximal and distal muscles in 53.8%, and the frequent cranial nerve involvement in children under 4 years of age. An unusually large percentage of 51.4% had an atypical presentation, i.e., a distal variant in 35.1%, pure motor variant in 13.5%, and pure sensory variant in 2.6% of the patients. Disease severity was milder compared to the adult population, with only 16.2% of children being unable to walk unaided at the disease nadir. Likewise, standard treatment approaches resulted in a favorable outcome in the majority of young patients, including complete remission in 51.4%, and minor residual deficits in 10.8%. Further studies are warranted in the pediatric population to facilitate timely diagnosis and inform optimal treatment approaches.

The review by Menon et al. focused on the treatment approaches for atypical CIDP. This is a timely article since CIDP variants differ not just in their clinical, pathological, and electrophysiological characteristics, but also in their variable response to conventional immunosuppressive agents effective against typical CIDP, and high quality evidence is lacking regarding best management options. Based on existing data, DADS has the phenotype of a symmetric, demyelinating sensory, length-dependent polyneuropathy and is frequently associated with paraproteinemia and anti-myelin associated glycoprotein (MAG) antibodies. While the management of idiopathic DADS (DADS-I) is similar to classic CIDP, DADS with an M protein (DADS-M) response is suboptimal while it may show a more favorable response to rituximab. On the other hand, MADSAM manifests as a chronic progressive demyelinating mononeuropathy multiplex which can evolve into a confluent pattern indistinguishable from CIDP. Evidence favors treating MADSAM with conventional immunomodulatory therapy although the response tends to be less satisfactory than in CIDP. A subgroup of patients presenting with purely sensory symptoms are known as pure sensory CIDP or chronic inflammatory sensory polyradiculoneuropathy (CISP) with presumed underlying pre-ganglionic pathology. Both respond well to first-line immunomodulatory therapy, particularly to IVIG, but relapse without maintenance. The pure motor CIDP resembles multifocal motor neuropathy with conduction block (MMNCB). However, previous reports of worsening with steroids have not been reproduced in recent studies, and a good response to both intravenous immunoglobulin (IVIG) and steroids is likely. Some of the focal forms of CIDP would defy an accurate classification but nevertheless respond to first-line therapies. Similarly most of the patients with focal forms of CIDP respond well to IVIG. Overall, most types of atypical CIDP are amenable to treatment with first-line immunomodulatory therapy but the response may be suboptimal compared to CIDP. There is increasing evidence for agents such as rituximab, especially in DADS-M and in cases that are refractory to first-line therapies, although further studies are needed to support optimal treatment algorithms in each variant.

Finally, the comprehensive review by Prof. Kira on combined central and peripheral demyelination (CCPD) associated with anti-neurofascin 155 (NF155) antibody provides a detailed description of diagnostic and treatment considerations for this very interesting CIDP variant. Overall, a small number of CIDP patients may harbor autoantibodies against molecules of the nodal region, such as nodal NF186, paranodal NF155, contactin 1 (CNTN1), or contactin-associated protein 1 (CASPR1). In most cases, the predominant IgG subclass is IgG4. These neuropathies present distinct features compared with antibody-negative CIDP, including poor response to IVIG. Neuropathology of the biopsied sural nerve shows detachment of Schwann cell paranodal loops from axons without macrophage infiltration or inflammation, partly attributable to IgG4 blocking protein-protein interaction without inducing inflammation. Anti-NF155 antibody-positive (NF155^+^) CIDP is unique because of the high frequency of subclinical demyelinating lesions in the central nervous system (CNS), owing to the expression of NF155 both in PNS and CNS paranodes. In NF155^+^ CIDP/CCPD hypertrophy of spinal nerve roots and cranial nerves, such as trigeminal and oculomotor nerves, and extremely high levels of cerebrospinal fluid (CSF) protein indicating nerve root inflammation are common. CXCL8/IL-8 and IL-13 levels are significantly higher in CSF, whereas IL-1β, IL-1ra, and IL-6 levels are lower in NF155^+^ CIDP than in NF155^−^ CIDP. Canonical discriminant analysis revealed NF155^+^ and NF155^−^ CIDP to be separable with IL-4, IL-10, and IL-13, the three most significant discriminators, all of which are required for IgG4 class-switching. Therefore, upregulation of both Th2 and Th1 cytokines and downregulation of macrophage-related cytokines are characteristic of NF155^+^ CIDP, which explains spinal root inflammation and the lack of macrophage infiltration in the sural nerves. At least in a Japanese cohort with NF155^+^ CIDP/CCPD, a higher prevalence of haplotypes HLA-DRB1^*^15:01-DQB1^*^06:02 was found compared with healthy controls, indicating a genetic predisposition with involvement of specific HLA class II molecules and relevant T cells in addition to IgG4 anti-NF155 antibodies in the mechanism underlying IgG4 NF155^+^ CIDP/CCPD. Further investigation of these immune and genetic aspects in other populations may shed more light on to the pathogenesis of this rare but challenging CIDP variant that responds well to rituximab treatment.

In summary, this collection of articles provides an important contribution to the better understanding of rare CIDP variants, including their clinical, electrophysiological, immunological, and genetic characteristics, and should be useful in increasing awareness and diagnostic accuracy for affected patients. Emerging novel biomarkers may further facilitate the distinction among these variants and support earlier differential diagnosis. Treatment considerations are increasingly studied and should be based on evidence, but the rarity of these disorders makes further collaborative study of optimal treatment approaches imperative, along with further research to develop more specific therapeutic protocols for each CIDP variant.

## Author Contributions

All authors listed have made a substantial, direct, and intellectual contribution to the work and approved it for publication.

## Conflict of Interest

The authors declare that the research was conducted in the absence of any commercial or financial relationships that could be construed as a potential conflict of interest.

## Publisher's Note

All claims expressed in this article are solely those of the authors and do not necessarily represent those of their affiliated organizations, or those of the publisher, the editors and the reviewers. Any product that may be evaluated in this article, or claim that may be made by its manufacturer, is not guaranteed or endorsed by the publisher.

